# Intention-driven functional electrical stimulation therapy using a wearable high-density electrode sleeve in chronic stroke: a case report

**DOI:** 10.1186/s12984-026-02051-1

**Published:** 2026-06-24

**Authors:** Ian W. Baumgart, Michael J. Darrow, Nicholas J. Tacca, Collin F. Dunlap, Samuel C. Colachis, Ashwini Kamath, Bryan R. Schlink, Philip T. Putnam, Joshua Branch, David A. Friedenberg, Lauren R. Wengerd, Eric C. Meyers

**Affiliations:** 1 Neurotechnology, Battelle Memorial Institute, Columbus, USA; 2 NeuroTech Institute, The Ohio State University, Columbus, USA; 3 Texas Biomedical Device Center, The University of Texas at Dallas, Richardson, USA; 4 Department of Bioengineering, Erik Jonsson School of Engineering and Computer Science, The University of Texas at Dallas, Richardson, USA

**Keywords:** Functional electrical stimulation therapy, Intention-driven electrical stimulation, Electromyography-driven electrical stimulation.

## Abstract

**Background:**

Functional electrical stimulation (FES) has been recognized for decades as a method to retrain the motor system after stroke. Benefits of FES rehabilitation can be enhanced by (1) combining FES with task-oriented therapy (i.e. FES therapy (FEST)), (2) timing FES with the user’s volitional motor intention and (3) incorporating multiple trained tasks, as opposed to high repetitions of single tasks. Using a novel wearable FES technology, we tested therapy incorporating these elements in two chronic stroke survivors.

**Methods:**

Our group has developed the NeuroLife Sleeve, a wearable forearm sleeve that contains a high-density grid of embedded FES electrodes, that may be controlled by an operator or by the wearer’s own electromyographic (EMG) signals. The system enables rapid software-based switching between stimulation configurations for different functional movements without repeated electrode repositioning. During eight weeks of FEST, intention-driven FES enabling multiple movements was delivered via operator control twice weekly and EMG control once weekly.

**Results:**

At the end of the therapy period, subjects A and B had both improved their scores: Box and Blocks Test (A: +5, B: +7), the Action Research Arm Test (A: +7, B: +12), the Fugl Meyer Upper Extremity section (A: +11, B: +9), and the 9-Hole Peg Test (A: 158 s, B: 54 s, both previously unable). Clinical improvements persisted during the 10-week follow-up period, during which FES exposure was reduced by more than 80%.

**Conclusions:**

This case report provides a proof-of-concept demonstration of the NeuroLife Sleeve to support FEST and preliminary evidence that intention-driven FEST may support motor recovery in chronic stroke survivors. The NeuroLife Sleeve is a novel device designed to deliver this therapy through the easily donned wearable sleeve interface, support for both operator-triggered and EMG-controlled FES paradigms, and efficient transitions between tasks with programmable FES placement and parameters. Because both control modes were used throughout the intervention, the relative therapeutic contribution of each cannot be distinguished. *Trial registration*: We retrospectively registered the trial with ClinicalTrials.gov (16/01/2024, NCT06207240).

**Supplementary Information:**

The online version contains supplementary material available at https://doi.org/10.1186/s12984-026-02051-1.

## Background

Each year, nearly 800,000 people in the United States suffer a new or recurrent stroke [1]. Unilateral weakness, or hemiparesis, affects over 60% of stroke survivors [2], and fewer than 20% of stroke survivors regain complete function in their affected arm despite months of rehabilitation [3]. These deficits in upper extremity function are profoundly disabling and significantly affect one’s ability to complete activities of daily living [4]. To reduce impairment after stroke, motor rehabilitation is the gold standard [5]. However, impairment persists for many patients, making novel efficacious interventions an urgent priority.

Functional electrical stimulation (FES), in which electrical stimulation is used to evoke functional movement, is widely used as an adjuvant in stroke rehabilitation [6]. Rehabilitation may be enhanced when FES is paired with additional complementary strategies [7]. These strategies include combination with task-oriented training (TOT), synchronization of stimulation with motor intention, and practice spanning a range of movements and tasks. Below, we describe the evidence supporting each of the three strategies.

Task-oriented therapy, in which patients perform goal-directed functional task practice, generates greater neuroplastic effects compared to non-functional motor rehabilitation [8, 9]. This type of training encourages active engagement from the patient, which is critical for driving neuroplasticity and optimal motor recovery [10, 11]. Furthermore, successful completion of tasks provides reward feedback which drives motor skill learning and reorganization of neural circuitry to support long-lasting recovery [12]. Popovic and colleagues [13, 14] have defined the concept of FES therapy (FEST) as FES-enabled task training where the recipient is instructed to attempt the movement and the therapist delivers concurrent FES [13–16]. Delivering stimulation controlled by the therapist, FEST was shown to be superior for upper limb rehabilitation compared to conventional occupational therapy alone in 8-week courses of spinal cord injury and stroke [13, 15, 16]. These studies demonstrate that FES can be feasibly combined with TOT to improve the functional benefits of therapy and motivates further study [7, 17].

Closed-loop patient control, rather than therapist-controlled FES, may further augment the feasibility and efficacy of FEST. Closer pairing of motor intention and FES is believed to enhance stroke recovery through principles of Hebbian neuroplasticity by synchronizing descending motor intent commands with the appropriate assisted movement and resultant ascending sensory activation [18, 19]. Electroencephalography (EEG)-based user control has been used to automatically pair motor intention with FES, generating upper limb functional improvement in chronic hemiplegic stroke survivors [20, 21]. In these studies, EEG was used to initiate single movements or timed sequences, but more continuous user control over FES may confer a greater sense of agency, which has been suggested to support adaptive neuroplasticity and longer term “carryover effects” of FES [22]. Electromyography (EMG) has been investigated as an intuitive and more robust control signal for intent-driven FES and FEST interventions [23–30]. EMG-controlled FES has been shown to drive corticospinal plasticity similarly to EEG-controlled [31], however optimal parameters have yet to be identified and hardware limitations of current EMG-controlled FES systems limit conclusions regarding its relative efficacy [26].

Using a variety of movements and tasks trained during FEST sessions is limited by currently available technologies. Clinicians using TOT commonly tailor the tasks and difficulty to the patient’s ability and need, and practice multiple tasks each rehabilitation session [32]. However, most intention-driven FEST systems, such as FIT-FES, MyndMove and recoveriX, use adhesive patch electrodes requiring manual, trial-and-error placement by FES-trained therapists for each movement, leading to lost therapy time for electrode repositioning [16, 27, 33, 34]. Wearable FEST systems, such as the Bioness H200, enable consistent electrode placement, but enable typically less than 4 functional movements [35]. An ideal FEST system will combine the flexibility to change stimulation locations and parameters quickly with the consistency of wearable electrode placement.

To solve these gaps in FEST technologies, our group has created the NeuroLife Sleeve with two control schemes to pair FES with movement intention: operator- and EMG-controlled. The NeuroLife Sleeve is a wearable forearm sleeve containing a high-density array of up to 160 embedded electrodes that deliver individually programmable electrical stimulation. The garment was designed to allow for range of motion during FEST, and a software graphical user interface (GUI) enables pairing with motor intention via either button press or EMG-controlled. Switching between stimulation configurations via GUI enables efficient transition between different grasps and functional movements without the need to reposition electrodes. The NeuroLife Sleeve technology enables a therapy program incorporating all of these key strategies: (1) task-oriented training, (2) intention-driven assistance, (3) efficient switching between FES-enabled movements to facilitate multi-task practice during sessions.

In this case report, we evaluated the feasibility of the NeuroLife Sleeve to support multi-movement, intention-driven FEST in individuals with chronic stroke as a proof-of-concept demonstration of the platform. Two participants with hand impairment used the wearable sleeve during task-oriented therapy sessions in which FES was paired with movement intention using either operator-triggered stimulation or EMG control. This study examined whether the system could practically deliver multi-movement, intention-driven FEST within a task-oriented rehabilitation framework.

## Methods

### Study participants

This case report focused on feasibility and system functionality. Two participants, pre-identified from related pilot work, were the first individuals contacted; both met all eligibility criteria and provided informed consent (Table S1). No additional individuals were screened or excluded. This study enrolled adults with upper limb hemiparesis and stroke-related hand impairment that interferes with the ability to complete activities of daily life. Inclusion criteria were: age ≥ 18 years, a diagnosis of stroke with residual upper-limb impairment that interfered with activities of daily living, the ability to follow three-step commands, and classification as Stage 1–6 on the hand subscale of the Chedoke McMaster Stroke Assessment. Exclusion criteria included participation in ongoing upper-limb rehabilitation, co-occurring neurological or neuromuscular disorders, implanted electrical devices (e.g., pacemakers), persistent pain ≥ 7/10 in the affected limb, and any medical or anatomical condition the investigator judged could compromise safety or data integrity. A complete list is provided in the Supplementary Materials. Data were collected as part of an ongoing clinical study being conducted at Battelle Memorial Institute that was approved by the Battelle Memorial Institute Institutional Review Board (IRB0828, IRB0779). All subjects provided written informed consent before participation, in accordance with the Declaration of Helsinki. This study was retrospectively registered on ClinicalTrials.gov (16/01/2024, NCT06207240).

### Materials

**Fig. 1:**
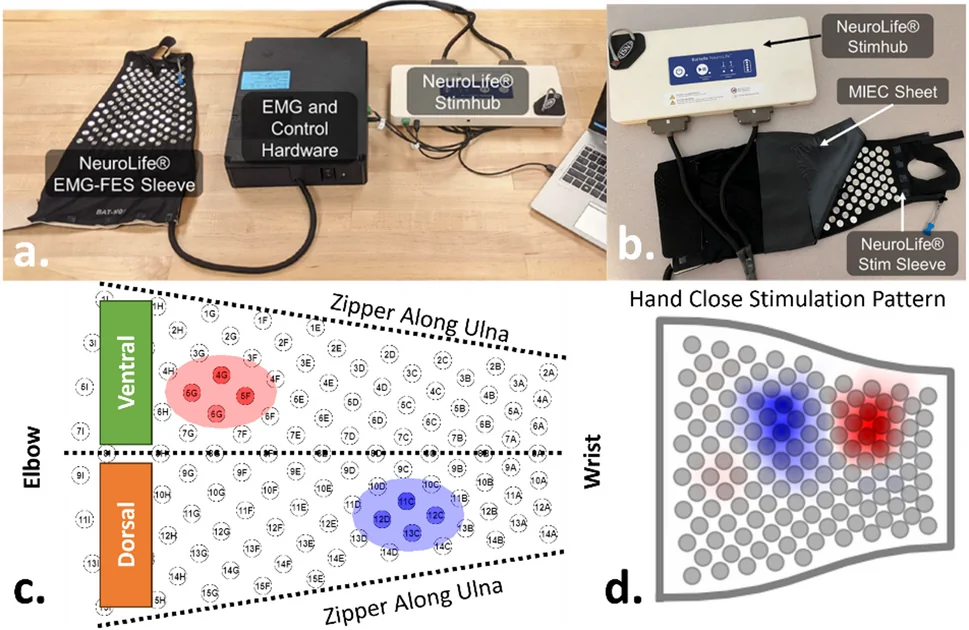
NeuroLife Sleeve systems. **a** EMG-controlled FES system (**b**) Operator-controlled FES system stimulator (NeuroLife StimHub) and sleeve with conduction enhancer sheet (MIEC sheet) (**c**) FES pattern calibration user interface with draggable and resizable anode and cathode ellipses. **d** Average Hand Close pattern configuration for subject 2B; blue indicates cathodic current amplitude, red indicates anodic current amplitude

Two NeuroLife Sleeve systems were used to deliver FES throughout rehab. The EMG-controlled FES system (Fig. 1a) can both sense movement intent via EMG and stimulate via FES, whereas the FES-only system (Fig. 1b) delivers FES via a GUI. Both systems consist of the fabric sleeve worn on the forearm, with a high-density array of 127 embedded stainless-steel electrodes and benchtop control unit(s) (Fig. 1a-b). The electrode array can be used for FES delivery and is also configured for EMG acquisition using 70 differential recording channels and a reference electrode positioned near the elbow when the sleeve is donned (see Fig. S5 for channel map). The electrodes are 12 mm in diameter, spaced 25 mm apart, and densely cover the forearm from elbow to wrist. In this study, the sleeve was donned and doffed by a zipper along the ulnar edge of the garment. Prior to donning, a conduction enhancing spray (Signaspray, Parker Laboratories, Inc.) was applied to the forearm and then the sleeve was placed on the forearm. A custom mixed ionic-electronic conductor (MIEC) enhancer sheet was placed between the electrodes and the skin to improve comfort during stimulation [36]. Impedance was not measured routinely throughout the study. However, testing of indicated typical skin–electrode impedance values of approximately 2–10 kΩ when measured at 1 kHz and 5 kHz.

Stimulation configurations for each movement (FES patterns) were calibrated through a software graphical user interface that allows for the independent selection of anode and cathode electrodes, stimulation frequency (20–50 Hz), and stimulation amplitude (up to 20 mA per electrode) (Fig. 1c). Stimulation amplitude was spatially modulated across the ellipses by a Gaussian-like function with tunable drop-off from center. All other stimulation parameters were kept constant, and asymmetric pulses were delivered (Phase 1: 500µs, Phase 2: 1000µs, inter-pulse duration: 200µs). Before the first therapy session, each subject underwent an initial calibration process to identify anode and cathode locations and amplitudes for each target movement. Stimulation frequencies were tailored to each movement in operator-controlled sessions but were fixed to 20 Hz in EMG-controlled sessions to facilitate EMG decoding. The combination of active electrodes and stimulation parameters are then fixed for each movement which together we term a FES pattern (Fig. 1d). Importantly, FES pattern calibration and EMG decoding were independent processes: FES patterns were manually configured by trained operators to evoke functional movements, whereas EMG decoding algorithms were separately trained and validated to detect the participant’s intended movement. Selection of active stimulation electrodes was performed manually through the GUI rather than through an automated search algorithm. During calibration, operators adjusted the location and size of virtual anode and cathode regions to produce smooth, task-relevant functional movements. Previously saved stimulation patterns served as starting points for subsequent sessions, and the high-density electrode array enabled rapid adjustment of active electrode locations to compensate for small shifts in sleeve placement following donning and doffing. Although these minor variations were not quantitatively analyzed, their presence underscores the need for adaptable stimulation configuration rather than strict reliance on identical electrode positioning across sessions.

Functional electrical stimulation (FES) patterns for each movement were configured using a standardized GUI that allowed trained operators to select and adjust stimulation parameters, including active electrode locations, current amplitude, and frequency. Each subject’s initial session began with loading previously saved stimulation templates corresponding to common functional movements, which served as standardized starting points. Operators then made real-time adjustments through the GUI to validate the evoked movement quality, with the goal of achieving smooth, task-relevant movements. Although the GUI facilitated efficient calibration and pattern switching, no formal usability assessment was performed, and its description here reflects system functionality rather than a validated usability outcome. These adjustments were made by trained study staff using consistent procedures across sessions. A single licensed occupational therapist oversaw FES delivery for participants throughout the study. Although stimulation configuration involved clinical judgment, this structured approach supported reproducible movement quality while allowing necessary individualization. No formal quantitative metrics were used to assess configuration consistency.

In the operator-controlled FES sessions, the FES-only version of the NeuroLife Sleeve system (Fig. 1b) was used. In these sessions, participants were instructed to attempt functional tasks normally, and the operator anticipated the subject’s intention and manually activated appropriate FES patterns via GUI buttons to assist with movement. In the EMG-controlled FES sessions, the EMG-controlled FES system (Fig. 1a) was used, which has bidirectional electrodes capable of acquiring EMG between stimulation pulses. EMG signals in the EMG-controlled FES system were acquired at 3 kHz sampling rate with a gain of 192 V/V using Intan Electrophysiology Amplifiers (Intan RHD2000, Intan Technologies, Los Angeles, CA) [37]. Decoding algorithms determined the intended movement (or rest) from EMG signals and triggered the appropriate FES pattern in real time, typically in 0.5s or less. A flowchart of the experimental setup of the EMG-controlled FES system is provided in the supplemental material (see Fig S1).

### Decoding algorithms

**Fig. 2:**
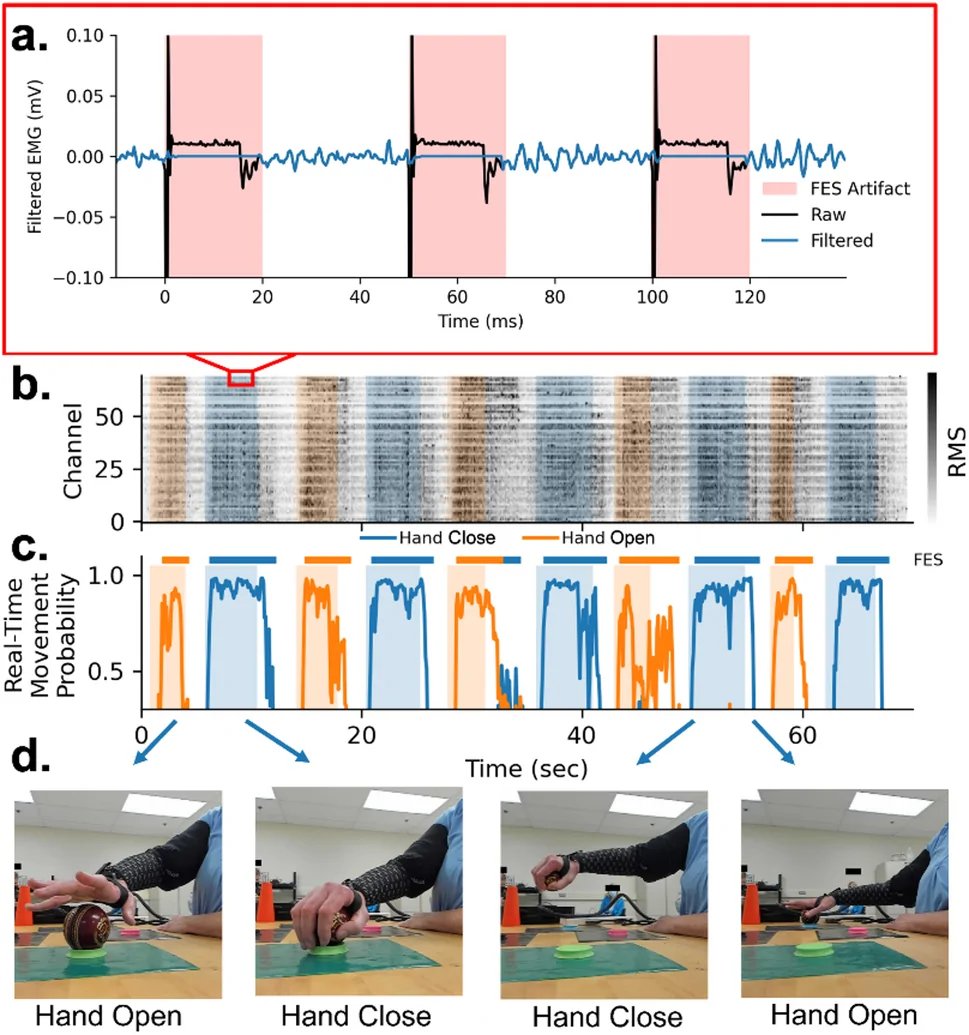
NeuroLife Sleeve operation. **a** A representative EMG signal including stimulus artifact (black) and after artifact removal filtering (blue) showing blanking during the artifact period (red shading). **b** RMS EMG amplitude over the 70 EMG channels. Shading indicates the movement observed by the operator. **c** Movement decoder probability. Bars over the plot indicate FES delivery based on decoder prediction. **c** FES-enabled movement used for afunctional task (transferring a ball)

To enable a participant’s volitional control of the FES, real-time classification algorithms were trained at each session using EMG signals to predict intended movements. Several minutes of data, broken into cued “blocks”, were collected at the beginning of each session for algorithm training. In each block, subjects were visually cued to perform isolated movements or to execute a functional task in steps. To label the functional task data, at most two functional movements were identified as components for each task, and labels were assigned to the component task step (e.g. Hand Open was labeled during the reaching phase and Hand Close was labeled during the grasp phase of the ball grasping task in Fig. 2c). These blocks were repeated with and without assistive FES delivered. EMG data were bandpass filtered (20–400 Hz, 10th order Butterworth filter) with a 60 Hz notch filter. Before filtering, EMG samples coinciding with known stimulation-pulse windows were blanked to remove stimulus artifacts, limiting their impact on RMS features (Fig. 2). A 20 ms blanking window was applied after each FES pulse, based on benchtop characterization of amplifier recovery time (~ 20 ms) to ensure artifact removal before EMG feature extraction. After blanking, adjacent signal segments were smoothly rejoined using cosine annealing prior to RMS feature extraction to minimize transient edge artifacts introduced by blanking. The root mean square (RMS) values for each channel were computed for 100ms non-overlapping bins and a 600-ms shift in cue labels was applied to account for the reaction time of the subject (Fig. 2a). Decoder training and inference were therefore performed on processed RMS features rather than raw EMG waveforms acquired during active stimulation. Neural network classifier models were trained on samples of four consecutive RMS bins and component movement labels for each task to be practiced in a session. The model input was a flattened array of bins and channels connected to two densely connected layers of 1000 and 500 nodes. A Softmax activation function was applied to the model outputs to provide prediction probability for each movement (Fig. 2c). A full description of the model can be found in the Supplementary Material. To test the performance of the decoding algorithms, an additional block of cued tasks was collected. If the computed bin-wise accuracy for the test block was below 80%, the classifier was iteratively retrained by adding the previous test block to the training set and collecting a subsequent test block. This accuracy threshold was determined empirically in prior work and used as a practical criterion to ensure reliable real-time FES control during therapy sessions rather than as a standardized benchmark for comparing decoding performance across movements. Including data collection and decoder training, the calibration procedures typically required approximately 15–30 min per session. Complete description of the real-time algorithms can be found in our previous publication [38].

All EMG decoding and FES control algorithms were implemented and executed on a standard portable workstation. Specifically, we used an HP ZBook Power 15.6″ G8 Mobile Workstation equipped with an Intel Core i7-11850 H @ 2.50 GHz processor, 32GB RAM, and an NVIDIA T1200 GPU running CUDA 12.2. All software components, including data acquisition, feature extraction, decoder training, and real-time control, were developed and run using Python 3.8.

### Experimental setup

During the 8-week intervention period, therapy was administered during three 2-hour sessions with the operator-controlled system used twice per week, and the EMG-controlled system used once per week. In these sessions all of the subject’s FES patterns were recalibrated from previously created patterns. In operator-controlled sessions, an operator matched FES to the subject’s intention during occupational therapist (OT)-guided therapy. At each session, the OT chose functional tasks (Table S2), graded them to an appropriate difficulty, and administered practice for approximately 20 min before moving on to a new task. Task selection and grading followed a framework grounded in established occupational-therapy principles—for example, progressively increasing task complexity, manipulating object size or resistance, and adapting hand-use requirements. A single licensed OT administered all sessions to ensure consistency in clinical style and decision-making. Although judgment was required to individualize tasks, the overall task categories, targeted movements, and grading strategies were standardized across participants. Detailed examples of task activities, their associated FES-enabled movements, and grading variations are provided in Fig. S2 and Table S2. Subjects were allowed time to rest between tasks and when requested to prevent fatigue. In EMG-controlled sessions, FES patterns were recalibrated and movement intention decoders were trained before therapy. Several therapy tasks involved single FES-assisted movements (e.g., grasping an object), whereas others required multiple sequential movements. In EMG-controlled sessions, only tasks incorporating Hand Open, Hand Close, or both (Hand Open and Hand Close) movements were used, and all movements were performed within the context of functional task practice rather than isolated motion trials. For instance, the cone-stacking task used both hand-opening and hand-closing patterns. During such tasks, participants switched between FES-enabled movements in real time via EMG-based intent detection, without manual reprogramming or operator input once the session began. Although decoders and stimulation patterns were recalibrated at the start of each session, the within-task switching itself was fully autonomous. TOT was administered by researchers trained by the OT in this study. The subject’s arm was inspected before donning and after doffing the sleeve to ensure there were no observable changes to the skin.

During the 10-week follow-up period, no task-oriented therapy was delivered; instead, participants attended ad-hoc sessions used for ongoing system development and engineering testing. The goal of these sessions was to collect data for advancement of EMG decoding algorithms and reliability of FES pattern calibration. FES patterns were applied intermittently (20–50 Hz, ≤ 20 mA per electrode, 1–3 s per activation), yielding an 84.4% reduction in weekly stimulation time for subject A and a 96.4% reduction for subject B compared with the intervention phase. Dose metrics for this period are summarized in Fig. S3.

### Clinical assessments

Clinical assessments were performed at the following timepoints: before the intervention (Pre), 4 weeks during intervention (Mid), 8 weeks immediately at the conclusion of the intervention (End), 2 weeks post-intervention (Post-2), 4 weeks post-intervention (Post-4), and 10 weeks post-intervention (Post-10). Clinical assessments included the Action Research Arm Test (ARAT), Upper Extremity Fugl Meyer (UEFM), Box and Blocks Test (BBT), and the Nine-Hole Peg Test (NHPT). Time limits were not imposed on the NHPT assessment, but subjects were allowed to discontinue the test if they felt unable to complete it. The Motor Activity Log (MAL) was completed at the end of operator-controlled sessions as a means to discuss strategies for incorporating hand use in real-world settings as part of the Transfer Package method [39]. These assessments were chosen to span the domains of the World Health Organization International Classification of Functioning framework in order to assess the holistic changes during and after intervention [40].

## Results

Two adults with upper extremity hemiparesis due to stroke were enrolled in the study. These subjects were both over 60 years of age and in the chronic phase of stroke recovery (> 2 years). Impairment of both subjects was classified as moderate at baseline assessment based on UEFM scores [41] (Moderate: 19 ± 2 to 47 ± 2 points, A: 28 points, B: 34 points). No serious unexpected adverse events nor device-related adverse events occurred during the study intervention or follow-ups.

### NeuroLife system enabled intention-driven FES with a variety of tasks

**Table 1:** Summary of clinical assessment scores at all timepoints

Assessment	Subject	Pre 0	Mid 4	Post 8	Post-2 10	Post-4 12	Post-10 18
ARAT MCID: 5.7	A	25	27 (+ 2)	32 (+ 7)*	32 (+ 7)*	34 (+ 9)*	39 (+ 11)*
	B	35	42 (+ 7)*	47 (+ 12)*	48 (+ 13)*	47 (+ 12)*	47 (+ 12)*
UEFM MCID: 5.25	A	28	33 (+ 5)	39 (+ 11)*	39 (+ 11)*	34 (+ 6)*	37 (+ 9)*
	B	34	41 (+ 7)*	43 (+ 9)*	43 (+ 9)*	41 (+ 7)*	42 (+ 8)*
BBT MCID: 5.5	A	13	15 (+ 2)	18 (+ 5)	22 (+ 9)*	-	23 (+ 10)*
	B	28	30 (+ 2)	35 (+ 7)*	32 (+ 4)	40 (+ 12)*	35 (+ 7)*
NHPT	A	Unable	234	158	105	116	97
	B	Unable	78	53.9	51.6	70	91
MAL - AOU MCID: 1.0	A	0.77	1.85 (+ 1.08)*	2.69 (+ 1.92)*	-	-	-
	B	1.35	2.35 (+ 1.00)*	3.13 (+ 1.78)*	-	-	-
MAL - QOM MCID: 1.1	A	0.85	1.96 (+ 1.11)*	2.54 (+ 1.69)*	-	-	-
	B	1.21	2.38 (+ 1.17)*	2.96 (+ 1.75)*	-	-	-

Assessment scores and change from Pre timepoint in parentheses. Changes exceeding the MCID are indicated by (*). Assessments not administered are indicated by (-). Missing entries were not possible to compute

Descriptive statistics of therapy are reported as median (inter-quartile range) for each subject. Subject A received an average of 145 (95-174) stimulations per session with an average therapy duration of 51.8 (44.0-74-7) minutes in each session, similar to previous FEST studies [20, 42]. Subject B received an average of 156 (123-189) stimulations per session with an average therapy duration of 49.5 (42.0-70.7) minutes in each session. Each intention-driven stimulation event lasted an average 2.0 (1.27-3.20) seconds for subject A and 1.86 (1.06-3.05) seconds for subject B. Over the course of the intervention, 16 task-oriented therapy activities were practiced with each subject. FES patterns were created to assist with 6 movements for subject A and 5 movements for subject B (Fig S4). An average of 2 (2-3) movements were used per session for both subjects in operator-controlled sessions. EMG-controlled sessions were restricted to Hand Open and Hand Close movements, both of which were used in every session. Within those EMG sessions, participants alternated between hand-open and hand-close stimulation in real time without therapist input, illustrating the task-level switching advantage of EMG control. Real-time EMG decoders were deployed only when offline accuracy on a held-out test block exceeded 80%, guaranteeing reliable FES control. In practice, all sessions met this performance threshold, and no sessions required cancellation or substitution with operator-controlled stimulation. Although we did not directly compare decoder accuracy with operator-triggered stimulation, every EMG-controlled session met this criterion and enabled successful task engagement, underscoring the practical usability of intention-driven control.

**Fig. 3:**
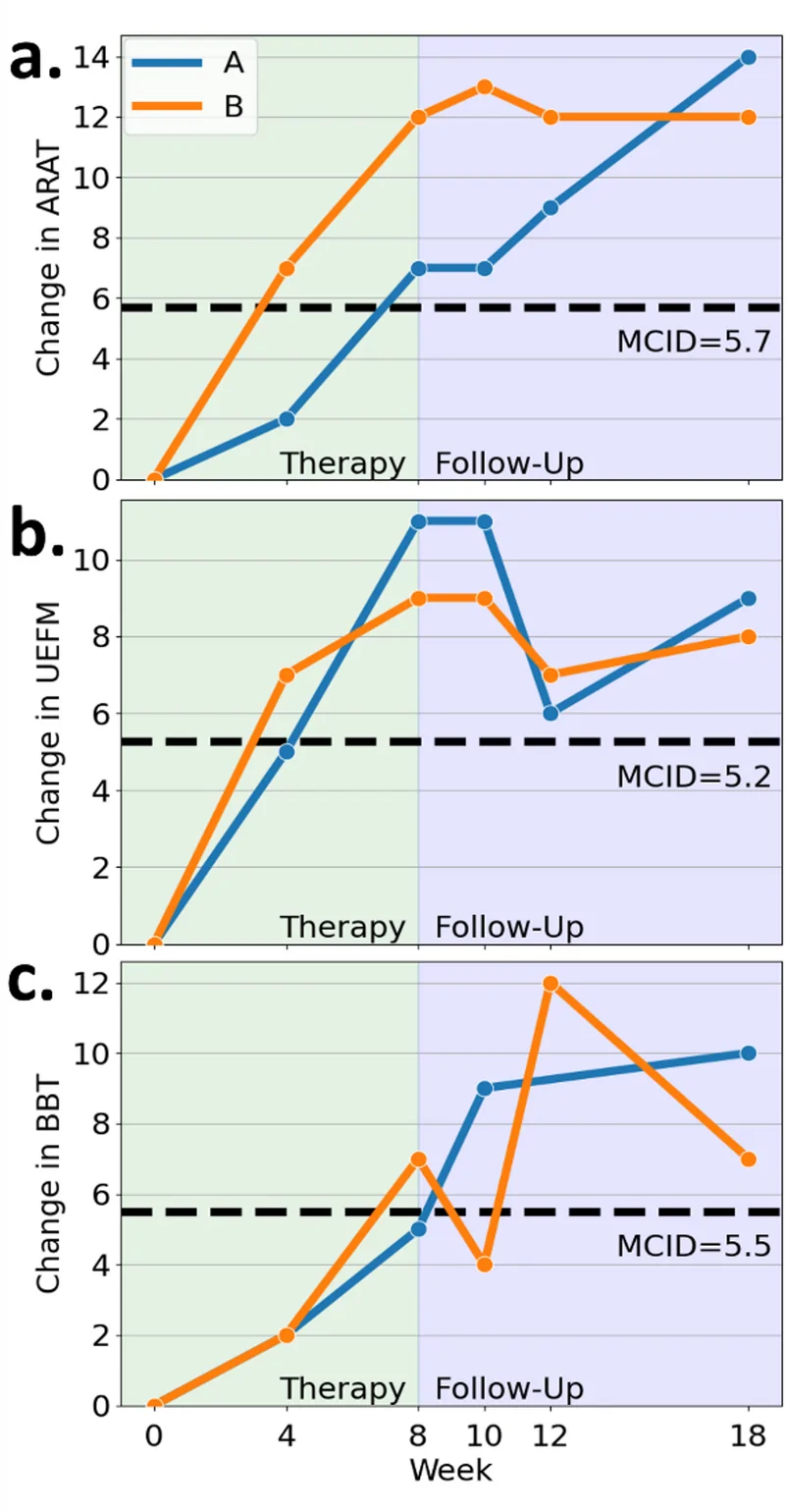
Change in motor recovery at the outcome timepoints. **a** Action Research Arm Test (ARAT). **b** Upper Extremity Fugl Meyer (UEFM). **c** Box and Blocks Test (BBT). Black lines indicate the minimal clinically important difference (MCID). Green shading indicates the therapy intervention period, and blue shading indicates the follow-up period

### Both subjects exceeded MCID thresholds on multiple assessments

At the conclusion of the 8-week therapy schedule, both subjects demonstrated improvements that exceeded the minimal clinically important difference (MCID) in at least two assessments [43–45] (Table 1; Fig. 3). ARAT scores improved beyond the MCID (A: +7, B: +12, MCID: +5.7). UEFM scores improved beyond the MCID (A: +11, B: +9, MCID: +5.25). BBT scores improved for both subjects and beyond the MCID for subject B (A: +5, B + 7, MCID: +5.5). 9HPT scores improved to 158 s for subject A and 53.9 s for subject B, whereas both subjects were unable to perform the test at the pre-intervention timepoint (Pre). Although the MAL was not an outcome measure for this study, the MAL scores improved above MCID for both Amount of Use (A: +1.69, B: +1.78, MCID: +1.00) and Quality of Movement (A: +1.92, B: +1.75, MCID: +1.10). The BBT for subject A was not administered at the 4-wk follow-up timepoint due to an injury that was determined unrelated to the study.

### Assessment scores were maintained during the reduced-dose follow-up period

The therapeutic improvements observed during the first 8 weeks were sustained during the 10-week follow-up period. During this time, subjects continued receiving FES at a reduced dose, constituting an 84.4% reduction for subject A and 96.4% reduction for subject B in mean weekly stimulation duration (Fig. S3). These findings suggest that clinically meaningful improvements may be maintained despite a markedly lower delivered stimulation dose. However, because participants continued receiving intermittent FES during this period, the follow-up phase should not be interpreted as a true no-treatment washout period, and maintained improvements cannot be attributed solely to the initial intervention. The ARAT and UEFM improvements remained clinically meaningful for both subjects during the 10 weeks following intervention (Fig. 3). Subject A demonstrated continued improvement on the ARAT assessment during the follow-up period.

## Discussion

In this study, two subjects underwent 8 weeks of intention-driven FEST 3 times per week. At each session, subjects received approximately one hour of FES-enabled practice with tasks using multiple functional movements. Both subjects showed clinically meaningful functional improvements throughout the intervention period, as shown by improvements in the ARAT, UEFM, NHPT, and BBT assessments. Clinical improvements persisted throughout the 10-week follow-up, during which FES exposure was reduced by 80%. The session duration and number of repetitions per session in this study were greater than those observed in other FEST studies [7, 46]. These results demonstrate feasibility of the NeuroLife Sleeve to deliver multi-movement, intention-driven FEST across multiple functional tasks in chronic stroke survivors.

These results build upon previous research on the effects of FEST supporting functional recovery. The intervention in this case report comports with recommendations for FEST administration: mandatory volitional effort from participants, electrical stimulation evoking functional task-related movement, and therapist guidance during task execution [7, 14, 17]. The duration (8 weeks) and weekly dose (~ 1 h, 3 times per week) of this intervention are also similar to previous studies of FEST for upper limb stroke rehabilitation [15, 17, 20, 42]. Previous studies of FEST for upper limb stroke rehabilitation have shown clinically meaningful improvements to upper limb function, similar to those shown in this case report [15, 16, 27, 35].

Pairing FES with motor intention is hypothesized to engage neuroplastic mechanisms of recovery. In case studies of EEG-controlled FEST with comparable durations and upper-limb tasks, similar improvements have been observed in ARAT and UEFM scores in chronic stroke survivors [20, 42]. In these studies, EEG was used to initiate a timed sequence of FES patterns, whereas in the FEST sessions here, operators and EMG decoders were used to control both the initiation and termination movements continuously to execute functional tasks. Jonsdottir et al. (2017) observed improvements to ARAT and UEFM scores, similar in magnitude to those reported here, in their chronic study subjects using the Myoelectric Controlled FES (MeCFES) system enabling continuous and proportional control of FEST [27, 34]. Our results are consistent with the hypothesis that closed-loop volitional control of FES may provide a greater sense of agency to the user and more consistent active participation, enhancing the neuroplastic and functional benefits of the therapy.

The improvements in ARAT and UEFM scores observed in this study are consistent with those reported in previous EEG-based FES interventions [20, 21, 42], suggesting that both modalities may facilitate functional recovery in chronic stroke. However, EMG-based control offers several distinct advantages. EMG signals provide a more direct and spatially resolved representation of peripheral motor intent, capturing descending motor output at the level of the muscle rather than upstream cortical correlates. This temporal and anatomical specificity allows for real-time control that more precisely aligns with volitional effort and movement execution. Prior literature suggests that this alignment between volitional effort and movement execution may enhance Hebbian mechanisms of neuroplasticity by synchronizing motor commands with afferent sensory feedback [18, 19]. From a practical standpoint, EMG-based systems also offer key usability benefits. They require less setup time, eliminate the need for conductive gels, and are more robust to environmental noise and signal non-stationarity compared to EEG. These attributes make EMG-based control more suitable for real-world and home-based deployment, supporting its potential for widespread rehabilitation use.

However, EMG-controlled FES has not shown clear superiority to other interventions in meta-review [26]. This may be due to sub-optimal, inconsistent parameters and hardware limitations of systems used to deliver EMG-controlled FES. Another inconsistency in EMG-controlled and intention-driven FES upper limb rehabilitation literature is the number of muscles, movements, and tasks targeted in interventional studies. Two recent randomized clinical trials showed improvements to upper extremity function with EMG-controlled FES practice of wrist and finger extension that were non-significant compared to cyclic FES [24] or conventional therapy alone [47]. These findings, taken with the significantly greater improvements compared to conventional therapy found by Thorsen et al. [48] may indicate that EMG-controlled FES is better applied for TOT than isolated movement practice alone.

Furthermore, more limited improvement with EMG-controlled FES interventions has been observed in subjects with more severe baseline upper limb impairment [26, 27, 47]. EMG control schemes require some volitional muscle activation remaining after injury to control FEST, which may make EEG control more feasible for more impaired users [20]. Stroke-related factors such as lesion location, corticospinal tract integrity, residual voluntary muscle activation, EMG signal-to-noise ratio, participant compliance with cued movements, and environmental interference may all influence EMG decoding performance and responsiveness to EMG-controlled FEST interventions; however, these factors were not independently quantified in the present study. Kwakkel et al. and Jonsdottir et al. note that EMG-controlled FES interventions were adapted for subjects with EMG signals insufficient for FES control by placing EMG electrodes on contralateral unaffected or synergistic muscles [27, 47]. While these enable therapy, reduced coupling of motor intention and FES sensory activation in this off-target control scheme may be suboptimal to drive neuroplastic effects. Subjects in the present case report were both categorized as moderately impaired (UEFM between 19 and 47 points) and in the chronic (> 9 months post-stroke) at baseline [41]. Our group has previously shown continuous EMG-based decoding of multiple movements using the EMG-only version of the NeuroLife Sleeve in severe chronic stroke survivors (UEFM hand subscore < 3), including scenarios without visible movement [49]. However, further studies are needed to determine whether similar results are possible with the additional complexity of decoding through stimulation, which allows for recording and stimulation through the same electrodes in this system. Although the use of shared electrodes for stimulation and EMG acquisition introduces a known risk of artifact contamination, our artifact-mitigation strategies and decoding-validation procedures helped ensure reliable performance. Real-time decoding was only used when the model demonstrated ≥ 80% accuracy in offline testing, serving as a practical quality-control measure. Furthermore, consistent user control of FES-assisted movements across sessions suggests that volitional EMG signals were successfully extracted despite the potential for residual artifacts. Future system refinements may incorporate hardware-level solutions such as amplifier blanking or alternative electrode-switching strategies to further improve signal quality during stimulation.

Finally, to facilitate integration with existing clinical practices, an intention-driven FEST system should readily allow for adjustment to task challenge and type throughout the session [32, 50]. In the *Practical Considerations for Therapist* section of Kapadia et al.’s review of FEST for reaching and grasping, 3 of 12 general procedure steps concern placement and calibration of patch FES electrodes, highlighting the complexity and importance of this aspect of setup [17]. The NeuroLife Sleeve is designed to facilitate repeatable placement of electrodes through a zipper closure aligned along the ulnar edge from the styloid process to the elbow, supporting approximate repositioning across sessions. Although the NeuroLife Sleeve lacks the spatial resolution of commercially available HD-EMG research grade systems (e.g., OT Bioeletronnica), it provides broader coverage across the entirety of the forearm with improved usability for use in a clinical setting. Recording EMG activity from all muscles of the forearm using these research grade systems is often infeasible in a clinical setting due to the complicated setup and number of high-density arrays required. Previous EMG-driven FES systems [27, 51] designed to improve upper limb motor function following neurological injury often use a small number of EMG electrodes placed over one or two target muscles. To avoid negative effects of stimulation artifacts, these EMG electrodes are not placed in the immediate vicinity of the stimulation electrodes. In contrast, the NeuroLife Sleeve provides a higher density array of electrodes to capture spatial muscle activity across the entire forearm. Furthermore, the system is able to record EMG from all electrodes of the array, regardless of the location of stimulation. Combined with the ability to quickly change active electrodes in the array and save and load previous FES patterns in the GUI, this system reduces the manual burden of electrode placement and recalibration.

### Limitations

While results from this case report are encouraging, several limitations should be considered. The small sample size (*N* = 2), heterogeneity of stroke pathology, and lack of a control group preclude statistical conclusions about efficacy or the mechanisms underlying the observed improvements. Accordingly, this study should be interpreted primarily as a proof-of-concept feasibility demonstration of the NeuroLife Sleeve platform and its ability to support multi-movement intention-driven FEST. Any interpretations beyond feasibility should be considered preliminary and require confirmation in larger, controlled studies. The primary aim of this study was to demonstrate technical feasibility and the potential for clinically meaningful improvement using the NeuroLife Sleeve system. Both participants were more than four years post stroke, reducing the likelihood that spontaneous recovery explains the improvements; however, a controlled study will be required to confirm therapy related effects.

Two different methods were used to estimate motor intention and trigger FES during the intervention. Operator controlled stimulation was used in two of three weekly sessions, while EMG controlled stimulation was used in the third due to limited availability of the prototype system. Because both control modes were employed throughout the study, the therapeutic contributions of each cannot be distinguished. In addition, EMG controlled sessions were limited to gross hand movements (hand open and close) to ensure reliable decoder performance.

The EMG decoding approach also required session specific calibration. Although both participants consistently achieved decoder accuracies above the predefined 80% threshold, new training data and validation were required at each session. This approach enabled adaptation to day-to-day variability in EMG signals but limits scalability and long-term deployment. Future work will focus on improving decoder robustness, reducing calibration time, and enabling more complex motor control.

Finally, interpretation of long-term outcomes is limited by the follow up period design. Although both participants maintained clinically meaningful improvements 10 weeks after the intervention, they continued to receive intermittent FES sessions during ongoing device development, which represents a confound. Consequently, the follow-up period should not be interpreted as a true no-treatment washout period, and the maintained clinical improvements cannot be attributed solely to the initial 8-week intervention. Increased voluntary use of the affected limb, reflected by higher MAL scores, may also have contributed to functional gains. Larger controlled studies will be necessary to determine optimal therapy dose and durability of treatment effects.

## Conclusion

In this case report, we present two chronic stroke subjects with improved upper limb function following 8 weeks of (1) FES during task-oriented therapy (2) controlled by user intention (3) spanning multiple movements. Intention-driven FES was delivered using the NeuroLife Sleeve system, a wearable sleeve with an embedded high-density electrode array. Outcome measures showed clinically meaningful improvements (above the MCID) over the intervention period, during which subjects received 1 h of therapy 3 days per week. Furthermore, clinically meaningful improvements remained above MCID during a reduced-dose 10-week follow-up period that included intermittent FES exposure. The intervention integrated well with standard clinical practice for task-oriented therapy. This study provides feasibility and preliminary evidence that intention-driven FEST interventions using wearable FES technology can facilitate clinically meaningful improvements.

## Supplementary Information

Supplementary Material 1.

## Data Availability

The data are available upon request.

## Abbreviations

FES: Functional electrical stimulation
FEST: Functional electrical stimulation therapy
EEG: Electroencephalography
EMG: Electromyography
TOT: Task-oriented training
GUI: Graphical user interface
MIEC: Mixed ionic-electronic conductor
RMS: Root mean square
OT: Occupational therapist
ARAT: Action Research Arm Test
UEFM: Upper extremity Fugl Meyer
BBT: Box and Blocks Test
NHPT: Nine-Hole Peg Test
MAL: Motor Activity Log
MCID: Minimal clinically important difference
